# *N*,*N*′-Bis[1-(thiophen-2-yl)ethylidene]ethane-1,2-diamine

**DOI:** 10.1107/S1600536811033691

**Published:** 2011-08-27

**Authors:** Abdullah M. Asiri, Abdulrahman O. Al-Youbi, Hassan M. Faidallah, Khalid A. Alamry, Seik Weng Ng

**Affiliations:** a Chemistry Department, Faculty of Science, King Abdulaziz University, PO Box 80203 Jeddah, Saudi Arabia; b Center of Excellence for Advanced Materials Research, King Abdulaziz University, PO Box 80203 Jeddah, Saudi Arabia; c Department of Chemistry, University of Malaya, 50603 Kuala Lumpur, Malaysia

## Abstract

Mol­ecules of the title compound, C_14_H_16_N_2_S_2_, have a centre of inversion in the middle of the –CH_2_–CH_2_– bond; the (C_4_H_3_S)(CH_3_)C=N–CH_2_– moiety is almost planar (r.m.s. deviation for non-H atoms 0.027 Å).

## Related literature

For a related transition metal adduct, see: Modder *et al.* (1995 ▶).
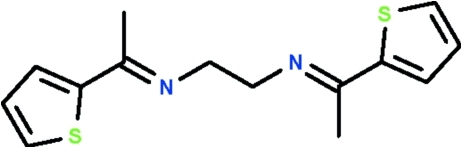


## Experimental

### 

#### Crystal data

C_14_H_16_N_2_S_2_
*M* *_r_* = 276.41
Monoclinic, 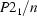

*a* = 5.5831 (3) Å
*b* = 9.3939 (4) Å
*c* = 12.9202 (5) Å
β = 95.342 (4)°
*V* = 674.68 (5) Å^3^
*Z* = 2
Mo *K*α radiation
μ = 0.38 mm^−1^
*T* = 100 K
0.25 × 0.20 × 0.15 mm

#### Data collection

Agilent SuperNova Dual diffractometer with Atlas detector
Absorption correction: multi-scan (*CrysAlis PRO*; Agilent, 2010 ▶) *T*_min_ = 0.912, *T*_max_ = 0.946
3036 measured reflections
1495 independent reflections
1244 reflections with *I* > 2σ(*I*)
*R*_int_ = 0.028

#### Refinement

*R*[*F* ^2^ > 2σ(*F* ^2^)] = 0.035
*wR*(*F* ^2^) = 0.090
*S* = 1.04
1495 reflections
83 parameters
H-atom parameters constrained
Δρ_max_ = 0.44 e Å^−3^
Δρ_min_ = −0.40 e Å^−3^

### 

Data collection: *CrysAlis PRO* (Agilent, 2010 ▶); cell refinement: *CrysAlis PRO*; data reduction: *CrysAlis PRO*; program(s) used to solve structure: *SHELXS97* (Sheldrick, 2008 ▶); program(s) used to refine structure: *SHELXL97* (Sheldrick, 2008 ▶); molecular graphics: *X-SEED* (Barbour, 2001 ▶); software used to prepare material for publication: *publCIF* (Westrip, 2010 ▶).

## Supplementary Material

Crystal structure: contains datablock(s) global, I. DOI: 10.1107/S1600536811033691/bt5618sup1.cif

Structure factors: contains datablock(s) I. DOI: 10.1107/S1600536811033691/bt5618Isup2.hkl

Supplementary material file. DOI: 10.1107/S1600536811033691/bt5618Isup3.cml

Additional supplementary materials:  crystallographic information; 3D view; checkCIF report

## Figures and Tables

**Fig. 1.**
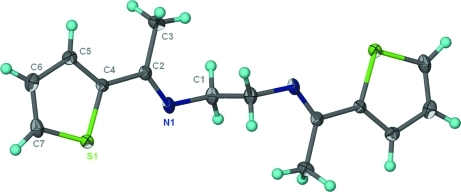
Anisotropic displacement ellipsoid plot (Barbour, 2001) of C14H16N2S2 at the 70% probability level; H atoms are drawn as spheres of arbitrary radius. The molecule lies on a center-of-inversion.

## supplementary crystallographic information

### Comment

A large number of transition metal adducts of Schiff bases derived by condensing ethylenediamine with a ketone have been reported; in these adducts, the ligand typically functions in a chelating mode. However, there are few studies on the title Schiff base (Scheme I), and only one crystal structure study has been reported (Modder *et al.*, 1995). The C_14_H_16_N_2_S_2_ molecule lies on a center-of-inversion (Fig. 1); the (C_4_H_3_S)(CH_3_)C═ N–CH_2_– moiety is planar, and the chain connecting the two aromatic rings adopts an extended zigzag conformation [C═N–C–C 88.1 (2)°].

### Experimental

Ethylenediamine (0.6 g, 10 mmol) and 2-acetylthiophene (0.7 g, 10 mmol) in dry benzene (50 ml) were refluxed in a Dean–Stark apparatus until no more water was collected (in about 2 h). The solvent was removed and the solid that separated was collected and recystallized from ethanol.

### Refinement

H-atoms were placed in calculated positions [C—H 0.95–0.98 Å, *U*_iso_(H) = 1.2–1.5*U*_eq_(C)] and were included in the refinement in the riding model approximation.

### Crystal data

**Table:**

C_14_H_16_N_2_S_2_	*F*(000) = 292
*M**_r_* = 276.41	*D*_x_ = 1.361 Mg m^−^^3^
Monoclinic, *P*2_1_/*n*	Mo *K*α radiation, λ = 0.71073 Å
Hall symbol: -P 2yn	Cell parameters from 1562 reflections
*a* = 5.5831 (3) Å	θ = 2.7–29.1°
*b* = 9.3939 (4) Å	µ = 0.38 mm^−^^1^
*c* = 12.9202 (5) Å	*T* = 100 K
β = 95.342 (4)°	Prism, colourless
*V* = 674.68 (5) Å^3^	0.25 × 0.20 × 0.15 mm
*Z* = 2	

### Data collection

**Table:**

Agilent SuperNova Dual diffractometer with Atlas detector	1495 independent reflections
Radiation source: SuperNova (Mo) X-ray Source	1244 reflections with *I* > 2σ(*I*)
mirror	*R*_int_ = 0.028
Detector resolution: 10.4041 pixels mm^-1^	θ_max_ = 27.5°, θ_min_ = 2.7°
ω scans	*h* = −5→7
Absorption correction: multi-scan (*CrysAlis PRO*; Agilent, 2010)	*k* = −12→9
*T*_min_ = 0.912, *T*_max_ = 0.946	*l* = −16→16
3036 measured reflections	

### Refinement

**Table:**

Refinement on *F*^2^	Primary atom site location: structure-invariant direct methods
Least-squares matrix: full	Secondary atom site location: difference Fourier map
*R*[*F*^2^ > 2σ(*F*^2^)] = 0.035	Hydrogen site location: inferred from neighbouring sites
*wR*(*F*^2^) = 0.090	H-atom parameters constrained
*S* = 1.04	*w* = 1/[σ^2^(*F*_o_^2^) + (0.0323*P*)^2^ + 0.5515*P*] where *P* = (*F*_o_^2^ + 2*F*_c_^2^)/3
1495 reflections	(Δ/σ)_max_ = 0.001
83 parameters	Δρ_max_ = 0.44 e Å^−^^3^
0 restraints	Δρ_min_ = −0.40 e Å^−^^3^

### Fractional atomic coordinates and isotropic or equivalent isotropic displacement parameters (Å^2^)

**Table:**

	*x*	*y*	*z*	*U*_iso_*/*U*_eq_	
S1	0.66665 (8)	0.89024 (5)	0.29153 (3)	0.01547 (15)	
N1	0.5827 (3)	0.68042 (16)	0.45253 (11)	0.0141 (3)	
C1	0.5756 (4)	0.56063 (19)	0.52477 (14)	0.0163 (4)	
H1A	0.5065	0.5926	0.5887	0.020*	
H1B	0.7412	0.5264	0.5446	0.020*	
C2	0.4204 (3)	0.77676 (19)	0.44907 (13)	0.0122 (4)	
C3	0.2062 (3)	0.7852 (2)	0.51239 (14)	0.0168 (4)	
H3A	0.0574	0.7795	0.4659	0.025*	
H3B	0.2102	0.8756	0.5503	0.025*	
H3C	0.2120	0.7060	0.5619	0.025*	
C4	0.4397 (3)	0.89283 (18)	0.37376 (13)	0.0113 (4)	
C5	0.2995 (3)	1.0123 (2)	0.35677 (14)	0.0144 (4)	
H5	0.1648	1.0330	0.3940	0.017*	
C6	0.3775 (3)	1.1021 (2)	0.27724 (14)	0.0163 (4)	
H6	0.3017	1.1893	0.2561	0.020*	
C7	0.5731 (4)	1.0482 (2)	0.23535 (13)	0.0171 (4)	
H7	0.6491	1.0930	0.1812	0.021*	

### Atomic displacement parameters (Å^2^)

**Table:**

	*U*^11^	*U*^22^	*U*^33^	*U*^12^	*U*^13^	*U*^23^
S1	0.0182 (3)	0.0134 (3)	0.0156 (2)	0.00002 (19)	0.00604 (18)	0.00080 (18)
N1	0.0170 (8)	0.0116 (7)	0.0135 (7)	−0.0028 (7)	0.0004 (6)	0.0025 (6)
C1	0.0195 (10)	0.0134 (9)	0.0154 (8)	−0.0006 (8)	−0.0007 (7)	0.0034 (8)
C2	0.0132 (9)	0.0120 (9)	0.0111 (8)	−0.0033 (7)	−0.0008 (7)	−0.0025 (7)
C3	0.0148 (9)	0.0213 (10)	0.0147 (8)	−0.0010 (8)	0.0030 (7)	0.0010 (8)
C4	0.0118 (8)	0.0118 (9)	0.0102 (8)	−0.0018 (7)	0.0006 (6)	−0.0013 (7)
C5	0.0131 (9)	0.0150 (9)	0.0151 (8)	0.0000 (8)	0.0007 (7)	−0.0015 (7)
C6	0.0185 (10)	0.0134 (9)	0.0156 (8)	0.0012 (8)	−0.0055 (7)	0.0013 (7)
C7	0.0239 (10)	0.0146 (9)	0.0123 (8)	−0.0052 (8)	−0.0006 (7)	0.0019 (7)

### Geometric parameters (Å, °)

**Table:**

S1—C7	1.712 (2)	C3—H3A	0.9800
S1—C4	1.7278 (17)	C3—H3B	0.9800
N1—C2	1.279 (2)	C3—H3C	0.9800
N1—C1	1.465 (2)	C4—C5	1.375 (2)
C1—C1^i^	1.523 (4)	C5—C6	1.428 (3)
C1—H1A	0.9900	C5—H5	0.9500
C1—H1B	0.9900	C6—C7	1.361 (3)
C2—C4	1.472 (2)	C6—H6	0.9500
C2—C3	1.513 (2)	C7—H7	0.9500
			
C7—S1—C4	92.09 (9)	H3A—C3—H3C	109.5
C2—N1—C1	120.31 (15)	H3B—C3—H3C	109.5
N1—C1—C1^i^	110.72 (18)	C5—C4—C2	129.41 (16)
N1—C1—H1A	109.5	C5—C4—S1	110.65 (13)
C1^i^—C1—H1A	109.5	C2—C4—S1	119.94 (13)
N1—C1—H1B	109.5	C4—C5—C6	112.95 (16)
C1^i^—C1—H1B	109.5	C4—C5—H5	123.5
H1A—C1—H1B	108.1	C6—C5—H5	123.5
N1—C2—C4	116.88 (15)	C7—C6—C5	112.08 (17)
N1—C2—C3	127.72 (16)	C7—C6—H6	124.0
C4—C2—C3	115.39 (16)	C5—C6—H6	124.0
C2—C3—H3A	109.5	C6—C7—S1	112.24 (14)
C2—C3—H3B	109.5	C6—C7—H7	123.9
H3A—C3—H3B	109.5	S1—C7—H7	123.9
C2—C3—H3C	109.5		
			
C2—N1—C1—C1^i^	88.1 (2)	C7—S1—C4—C5	0.05 (14)
C1—N1—C2—C4	−179.53 (15)	C7—S1—C4—C2	−179.44 (14)
C1—N1—C2—C3	−0.6 (3)	C2—C4—C5—C6	179.10 (17)
N1—C2—C4—C5	−176.62 (18)	S1—C4—C5—C6	−0.3 (2)
C3—C2—C4—C5	4.3 (3)	C4—C5—C6—C7	0.5 (2)
N1—C2—C4—S1	2.8 (2)	C5—C6—C7—S1	−0.5 (2)
C3—C2—C4—S1	−176.34 (13)	C4—S1—C7—C6	0.25 (15)

Symmetry codes: (i) −*x*+1, −*y*+1, −*z*+1.
